# Film, observation and the mind

**DOI:** 10.1177/09526951241235800

**Published:** 2024-03-20

**Authors:** Bonnie Evans, Janet Harbord

**Affiliations:** Queen Mary University of London, UK; Queen Mary University of London, UK

**Keywords:** film, history, neurology, neuroscience, psychology

## Abstract

This special issue considers the significance of film to the establishment and development of scientific approaches to the mind. Bonnie Evans explores how the origins of film technologies in 1895 in France encouraged a series of innovative collaborations, influencing both psychological theorisation, and new filming techniques. Jeremy Blatter explains how Harvard psychologist Hugo Münsterberg created early films specifically designed to engage audiences using psychological tactics. Scott Curtis’ article examines how Yale psychologist Arnold Gesell was able to extract scientific data from a film. Felix Rietmann’s article explores a collection of infant observation films from the 1930s and 1960s and how they developed unique narratives of mothers’ engagement with their children that did not necessarily match up with dominant scientific theories. Janet Harbord's article considers how a trilogy of films made at the Maudsley Hospital in the 1950s engaged with innovative film-making techniques that captured behaviour as discrete units. Seth Watter further examines how William S. Condon's use of the unique technology of the Bell and Howell 173BD projector in the 1960s created new understandings of human behaviour that could not have been predicted in advance, and which were highly influenced by the technology itself. Finally, Des O’Rawe explores how radical approaches in both anti-psychiatry, and documentary film-making in the 1960s created new opportunities for audiences to engage with different psychological states. All of these developments in film and psychology continue to influence understandings in both these fields to the present day.

The history of the mind sciences has often been told with a focus on written materials, such as publications of research findings and case studies that have privileged language and narrative. Yet, recently, there has been a growing interest in film as an important medium through which scientific knowledge in these areas has historically been propagated and co-produced, heralded by some as a visual turn in the medical humanities (Whitehead and Woods, 2016). This special issue of the journal brings together leading contemporary scholars in the history of film and the human sciences to consider the significance of the medium to the establishment and development of neurology, psychology, psychoanalysis, psychiatry, and related disciplines. Commenting on the history of science broadly, Oliver Gaycken has argued that the study of film provides many opportunities for new insights, while cautioning against an approach to visual documents ‘as transparent representations to be mined for content’ (Gaycken, 2015: 7). A common approach across the articles in this issue is an attempt to move beyond the study of film as merely the documentation of clinical activity, and an attempt to consider it as a truly dynamic object that has generated new techniques and practices that have shaped the clinical or experimental setting in which it has operated.

Recent work in the history of the mind sciences has explored the importance of clinical observations, and statistical surveys, in generating shared conceptual models of psychological norms and variances (e.g. Delille and Lezé, 2023). These have built on work on the history of statistical surveys and epidemiological studies to establish ‘norms’ within psychological modelling (e.g. Hayward, 2014; Rose, 1999; Shorter, 2015; Thomson, 2006). These studies have played an important role in locating diagnoses that have achieved importance within contemporary psychological research, and articulating the historical locations where these have originated. Moreover, this work has highlighted the importance of observational methods in creating shared consensus on both diagnoses and theoretical approaches, building on important work on the history of mental symptoms (Berrios, 1996). In fact, the creation of this volume was motivated by the realisation that work in this field could also benefit from an engagement of the longer history of film, and its transformational role in generating new perspectives on typical human actions and behaviour (Doane, 1999; Evans, 2024; Harbord, 2016; Landecker, 2006).

The role of photography in generating new perspectives in the mind sciences has been explored in work from the 1990s, and this has been influential across the disciplines of art history and film studies. Georges Didi-Huberman's early work on Albert Londe’s photography at La Salpêtrière argued for the central importance of photography in establishing the object of hysteria, and the book set a new benchmark for the analysis of images of hysteria and the female body (Didi-Huberman, 2003[1982]). Similarly, the work of Sander Gilman raised important questions about the role of visual culture in shaping scientific modelling in the brain and mind sciences, as well as broad cultural understandings of illness (Gilman, 1982). Photography, more than film, has been amenable to this analysis of the importance of visual culture in influencing understandings of illness and criminality within biopolitical analyses. Film, in contrast, presents a more heterogeneous set of operations, techniques, texts, and contexts that has perhaps been harder to locate within this framework.

The role that films have played in the history of the mind sciences took longer to materialise, but Alison Winter's pioneering work on the use of film to promote barbiturate ‘truth serums’ in psychiatric research and treatment was notable in establishing new approaches to the study of film. Her work demonstrated the important role played by films in supporting psychiatric research methods and conveying scientific findings (Winter, 2004). Winter, and the film historian Tom Gunning, argued that it was important to make psychological films available online, with the Internet generally increasing accessibility of films for the purposes of research. The medical historian Edgar Jones then demonstrated that film played an important role in advocating the work of specialists in ‘shell shock’ and ‘war neurosis’ during World War II (Jones, 2014). In 2016, the *Journal of the History of the Neurosciences* published a special issue on ‘Cinema and Neuroscience’ that explored neurological films from across Europe and their role in establishing scientific approaches in neurology (Aubert, 2016). Andreas Killen's *Homo Cinematicus* set new ground, exploring the importance of film in shaping all human sciences in Germany in the early 20th century (Killen, 2017). Anna Toropova has also recently examined the use of film in Soviet psychotherapeutic treatment, revealing the complex ways that film was employed as both observational science, but also therapeutic method in the first half of the 20th century (Toropova, 2022). Recent publications in this journal, including Tim Snelson and William Macauley's edited volume on the psy-sciences in the long 1960s and Katie Joice's article on cinematic microanalysis in the study of mothers and their children from the 1940s to the 1960s, have extended the study of film and psychology into the post-war period (Joice, 2021; Snelson and Macauley, 2021), as has Bonnie Evans’ latest article in *History of Psychiatry* on how film influenced universal approaches to international child development studies in the 1950s (Evans, 2024).

From the perspective of film studies, landmark accounts of the ways that medical film spanned both scientific education and entertainment can be found in the work of Lisa Cartwright and Tom Gunning (Cartwright, 1995). Tom Gunning's essay on the cinema of attractions (Gunning, 1986) established the period of early cinema as a heterogeneous (and, one might add, interdisciplinary) fascination with visual culture and its enchanting power, a period when science was matched by simple human engagement. Cartwright's *Screening the Body* (1995) challenged the conventional narrative in film history that commercial cinema was born out of early scientific experimentation and broke away from this in 1895. On the contrary, Cartwright argued, scientific film culture continued to influence, and dialogue with, commercial cinema throughout the 20th century. This was propagated via the question of ‘what constituted “life” for scientists and the lay public’. Motion studies designated the mechanics of human life and cinema, in its capacity to record and document, and also gave it the potential to preserve life (ibid.: 4). The work of Mary Anne Doane and Hannah Landecker has also demonstrated how early film created new visual typologies or means of viewing the world that shattered reliance on the written word and introduced the dimension of movement into understanding processes in the life sciences (Doane, 1999; Landecker, 2006).

Beyond the parameters of early cinema, however, the discipline of film studies has only tentatively included medical films within its framework. The focus on the hermeneutics of fiction film and ethnographic documentary has generally created a lacuna where clinical (and science) films reside. Only relatively recently have films of this nature entered the discipline as a marginal presence gathered together under the headings of ‘useful film’, or ‘films that work’, a subgenre at the edges of the history of the discipline and not specifically engaging medical history (Acland and Wasson, 2011; Hediger *et al.*, 2023; Vonderau and Hediger, 2009). In writing into or from the disciplinary histories of film and psychology, we intend to create a presence within film studies for the recognition of film's role in shaping the psychological sciences, and for the history of psychology to find new ways to investigate how the specific affordances of film staged, configured, and interacted with the events recorded and interpreted. This volume seeks to question the ways in which films were put to work in medical contexts, and how they often went against the grain of their commercial remit. The capacity to slow a film down exponentially, to watch film as both a sequential movement and as a series of still frames, to break down (anatomise) motion, offered new ways to understand human life in general. Whether film created an image of a body in motion or a number of sperm gametes, it generated new techniques for understanding human life that influenced psychological reasoning. When considering questions of objectivity, the fact that film facilitates the visualisation of matter (and activity) that the eye cannot see is important. Film prosthetically extended human capacity, and we can therefore revise film's ontological status from that of empirical tool of inscription to that of agent, co-constituting the object of medical investigation.

As discussed by Bonnie Evans in this volume, the development of film technologies in France at the end of the 19th century coincided with important developments in the mind and brain sciences. The use of film as a scientific method to capture atypical bodily movements for subsequent analysis was a technique that supported the fields of both neurology and psychology as fledgling fields of enquiry. As Lisa Cartwright has argued, neurological films appeared in stark contrast to the efficient bodies of workers documented in Frank and Lillian Gilbreths’ time-motion studies of labour efficiency. The ticking, twitching, rocking bodies of neurological patients stripped movement of its functionality and ‘the pathological body engages in acts that have nothing to do with purpose’ (Cartwright, 1995: 53). The recordings of these patients thus brought to light another feature of visual documentation: visual recordings are dependent not only on the immediate context of their production, but the wider network into which they acquire valence. For example, the Italian neurologist Vincenzo Neri filmed patients in his clinic and then revisited the material through techniques of frame enlargement and decomposition of the film into units. Evolving what Vanone, Lorusso, and Venturini name a ‘clinical semiotics’ through the use of film, Neri scrutinised the negative stock to isolate the moment in which the patient's pathology emerged (Vanone, Lorusso, and Venturini, 2016). Neri's embrace of film, influenced by the chronophotographic techniques of Etienne-Jules Marey, demonstrated that a filmed body acquired meaning through a complicated system of interpretation.

Another moment when film co-constituted the object of enquiry and shaped the research agenda of neurology and psychology was the interwar period, which saw the growing genre of child development studies. By the early 1920s, as child psychology gained strength as a discipline, the filmic apparatus was crucial in presenting the figure of the child as the object of study. Evans explains how the renowned French scientist Jean Comandon teamed up with film-maker Charles Pathé in 1907, and although their early collaborations with neurologists had focused on adult movement disorders, in 1922 they collaborated with Éduoard Claparède, director of the Psychological Laboratory in the Faculty of Sciences at the University of Geneva, to produce the first comprehensive film on psychological testing methods for children. Claparède was one of the leading child psychologists of the period, and his work was highly influential in establishing international collaboration among scholars to share methods and findings on child development (Depaepe, 1987).

Jeremy Blatter's article explores early collaborations in films and psychology from a different perspective, exploring the work of Hugo Münsterberg, the German-born psychologist who succeeded William James as director of the Harvard Psychological Laboratory in the last decade of the 19th century. Münsterberg also wrote what became known as the first iteration of film theory, *The Film: A Psychological Study* (1970[1916]). Uniting these two interests, in 1916 Münsterberg created a series of short films for cinema that were ‘tests’ to engage the audience psychologically. The ‘Testing the Mind’ series of films was a popularisation of psychology intended to transform passive audiences into active participants, yet the films themselves were lost. Blatter's contribution explores a new collaborative method of reconstructing historical psychology films from the contextual materials that have been archived, offering fresh perspectives on the relationship between psychology and early cinema.

As Scott Curtis has argued, the use of film in the study of children in the United States during the mid 1920s was part of a wresting of child observation studies from amateur, and largely female, ethnographers who produced diary accounts of their observations by an increasingly professionalised and funded (male) researcher. The demand for a scientific mode of observation was met by technical capacity of film to record without imposition on the child. Curtis drew attention to Gessel's famous quote that ‘cinema analysis is a form of biopsy which requires no removal of tissue from the living subject’ (Gesell, Amatruda, and Thompson, 1934: 22). Gesell's visual documentation of children represents one of the most extensive uses of film-as-research from the first part of the century. He famously enlisted Pathé Review, a newsreel production company, to film his work with a series of children as they engaged in various quotidian tasks. He extracted stills from the film that he called ‘action photographs’, as illustrations in what was to become a landmark publication, *The Mental Growth of the Pre-school Child*, and his later study, the monumental *Atlas of Infant Behaviour* (Curtis, 2011; Gesell, 1925, 1934).

Curtis’ article in this issue, ‘Behavior Takes Form: Tracing the Film Image in Scientific Research’, turns the evidential nature of film on its head by asking the question ‘How does one extract data from a film frame?’ The method that Gessel used, which was not untypical among researchers of the interwar period, was a form of intermediality: the tracing by hand of film frames. The practice served several purposes. Tracing the contours of a child's image was a means by which the researcher became familiar with the material. The line drawings also simplified the image, paring back the complexity of motion and context to a form that was a distillation of child behaviour. Curtis argues that the decontextualization of the child effected by the act of outline tracing, denied their individuality, producing instead an ideal image for the research. In place of singularity, the drive was toward typicality, the presentation of evidence that would confirm the validity of the research agenda.

From the opposite perspective, Felix Rietmann’s article explores a little-known collection of infant observation films made by Margaret Fries, Sybille Escalona, Mary Leitch, and other female child development specialists and psychologists from the 1930s up until the 1960s in which character development was paramount. Rietmann argues that these films presented very different narratives to films of ‘maternal deprivation’ made by psychoanalyst John Bowlby, whose BBC films with James Robertson were known internationally. Fries’ films explored character development in a cohort of children from birth to adolescence, bringing different perspectives on the way that film could capture individual personality. Fries’ other films on the experiences of early motherhood, and the responses of infants to their environment, present fascinating early attempts to film emotional states, catering to a growing interest in observational studies across child development specialists internationally. Rietmann argues that this group of women researchers shunned generalist explanations for emotional development in favour of specific observational studies: infants and children were the point of focus with only a vague presence of the mother in an attempt to recreate the thought processes of young children. Rietmann’s work thus reveals the wider cinematic landscape of psychoanalytic thought from the 1930s to the 1960s, giving a new perspective on film production in this period and revealing multiple sites through which we can understand the influence of psychoanalytic theory.

Film's reputation as an evidentiary medium in clinical and scientific work has focused critical attention on two filmic aspects: the context of recording and the film image. The study of post-production, however, elicits different questions arising from how medical films have been cut and joined in the process of editing. Janet Harbord's article offers one such account of a trilogy of films made in the children's ward at the Maudsley Hospital, London, in the late 1950s by the child psychiatrist and psychoanalyst Elwyn James Anthony. The central film, *Aspects of Childhood Psychosis*, documents the behaviour of children as they move about the institutional space, the camera focussing on that which appears errant or atypical in the realm of play. The means by which the film is assembled in post-production, however, crafts these recordings to give emphasis to short bursts of energetic and often repetitive behaviour; presented without contextual information, the quality of the children's movements is, from a contemporary perspective, GIF-like. Post-production is the facility through which the children's behaviour is parsed into modular units similar in type but disconnected from each other, evidencing a form of non-relationality. This in turn prepares the way for the diagnosis of autism as a condition of social isolation and empathy deficit. The filmic documentation of interpersonal communication in the field of kinesics during the post-war period, it is argued, produces the positive image of social interaction, with autism emerging as its negative.

The epistemological power of the film apparatus is also central to Seth Watter's contribution examining the psychologist William S. Condon's use of the Bell and Howell 173BD projector in his study of human behaviour during the 1960s. Condon was influenced by kinesics, particularly the project *The Natural History of an Interview*, involving a group of researchers in the US working collaboratively to study materials from the disciplines of anthropology, linguistics, and psychoanalysis. Condon's focus placed rhythm at the centre of acts of communication, demonstrating an unconscious synchronicity between speaker and addressee that, he argued, evidenced the central nervous system co-ordinating a unified somatic response. Watter's analysis is given to Condon's method, his practice of cranking a film projector by hand in the effort to produce short (five-second) sequences that were units of a synchronic dance. The researcher's replay of audiovisual materials offers a new understanding of the synchronization of speech and body movements within and between individuals in acts of communication. Watter's point is that Condon's work demonstrates that the ‘handling of definite instruments’, or cultural techniques that are embodied, produces a form of knowledge the purpose of which cannot be known in advance.

Finally, the presence of psychiatry in documentary film is the topic of Des O’Rawe's reflection on two films from the 1960s that explore approaches in psychiatric treatment. In this account of film and psychiatry, the contextual features of the anti-psychiatry movement, radical documentary practice, and new forms of film production equipment are brought to the fore. The post-war period, argues O’Rawe, witnessed the advent of a new mobile film culture facilitated by portable recording equipment, augmenting a series of movements, manifesting as free cinema in the UK, direct cinema in the US, and *cinema-vérité* in France, that engaged directly with the concept of madness. The political features of this new cinema were crafted through a visual language in which observation gave way to participation as the film-maker enters the frame of representation. The period in question is one in which a range of artistic influences, practices, and interventions were brought to bear on the subject of psychological illness, widening the opportunity for public engagement with such experiences.

The range of themes, practices, and historical periods in this special issue of the journal bring a number of historical spotlights on, and epistemological models of, the relationship of film to observation and the mind. The editors hope to encourage new work that further integrates film studies with the history of science, to generate new understandings of film, observation, and the mind since the late 19th century.
